# Development and implementation of a nurse-led clinical decision support tool for urinary tract infection

**DOI:** 10.1017/ash.2026.10772

**Published:** 2026-07-06

**Authors:** Lei A. Qin, Marguerite Balasta, Alison Purcell, Cara Curley, Rani Patel, Nathanael Koelper, Rebecca Hamm Feldman, Shafinaz Akhter, Lily A. Arya

**Affiliations:** 1 University of Pennsylvania Perelman School of Medicine, USA; 2 Obstetrics and Gynecology, University of Pennsylvania Perelman School of Medicine, USA

## Abstract

**Objective:**

Ordering urine cultures in the absence of urinary symptoms contributes to unnecessary antibiotic prescribing for urinary tract infection (UTI). The aim of this study was to develop and implement an electronic health record (EHR)-integrated, nurse-led clinical decision support (CDS) tool for UTI symptom triage to support outpatient antibiotic stewardship.

**Design:**

Exploratory sequential mixed-methods study.

**Setting:**

Urban academic family medicine practice.

**Participants:**

Eleven nurses.

**Methods:**

In the qualitative phase, workflow mapping and semi-structured interviews informed development of an EHR-integrated CDS consisting of a symptom score calculator and triage algorithm. In the quantitative phase, implementation was evaluated using a pre–post design with interrupted time series analysis over 15 months. Implementation outcomes included adoption, fidelity, and usability. Clinical outcomes included symptom documentation, urine testing practices, and antibiotic prescribing. Safety outcomes included pyelonephritis within 30 days.

**Results:**

Qualitative analysis identified four barriers: incomplete symptom documentation, reflexive urine testing, medicolegal concerns about missing pyelonephritis, and patient pressure for antibiotics. The CDS was adopted by all nurses, with 77.8% fidelity and high usability. Documentation of ≥2 UTI-specific symptoms increased at implementation with sustained improvement (slope + 2.4%/month; 95% CI, 0.1–4.7). Urine cultures without microscopy showed sustained reduction (slope −5.5%/month; 95% CI, −7.9 to −3.2). Antibiotic prescribing for asymptomatic bacteriuria decreased (20% to 3%; *P* = .003) with no increase in pyelonephritis.

**Conclusions:**

Nurse-led, EHR-integrated clinical decision support for UTI triage was associated with sustained improvements in symptom documentation, reduced unnecessary urine culture ordering, and decreased antibiotic treatment of asymptomatic bacteriuria without compromising safety.

## Introduction

Urinary tract infections (UTIs) are among the most common bacterial infections, accounting for more than 10 million ambulatory visits annually in the United States.^1,2^ Antibiotics are prescribed in over 80% of UTI-related encounters,^3^ yet an estimated 40%–50% of prescriptions are unnecessary that is prescribed when not clinically indicated.^4–6^ This evidence-practice gap contributes to adverse drug events, antimicrobial resistance, and more than $3.5 billion in annual healthcare costs.^7^ Despite most UTI antibiotics being prescribed in outpatient settings, stewardship efforts have largely focused on inpatient care and antibiotic selection rather than whether antibiotics are necessary.

Increasing evidence suggests that 40%–50% of uncomplicated UTIs improve without antibiotics, while progression to pyelonephritis is uncommon (1%–1.6%).^8,9^ Despite this low risk, concern about missing serious infection drives precautionary testing and antibiotic prescribing. Patients may attribute nonspecific symptoms such as cloudy or foul-smelling urine to UTI, while clinicians may order testing or prescribe antibiotics without systematically assessing UTI-specific symptoms, leading to treatment of asymptomatic bacteriuria for which antibiotics are not indicated.^6,10–12^

Structured symptom assessment during initial triage may help differentiate patients requiring diagnostic testing from those who can be safely managed with supportive care. Recent expert consensus supports symptom-based triage algorithms for outpatient UTI, recommending empiric treatment without testing for women with classic cystitis symptoms and urinalysis with reflex culture for those with resistance risk factors.^11^ Positioned between patients and prescribers, nurses are well suited to triage symptoms and deliver stewardship-aligned education yet remain underutilized in outpatient UTI stewardship. Electronic health record (EHR)-embedded clinical decision support (CDS) can operationalize this approach by standardizing symptom assessment and prompting systematic evaluation before initiating testing or antibiotics.

Our aim was to develop and implement an EHR-integrated, nurse-led clinical decision support (CDS) tool for UTI symptom triage to support outpatient antibiotic stewardship in primary care. We evaluated implementation outcomes (reach, adoption, fidelity, usability) and clinical process and patient safety outcomes.

## Methods

### Study design and setting

We conducted an exploratory sequential mixed-methods study consisting of a qualitative phase to develop a nurse-led, EHR-integrated UTI CDS tool, followed by a quantitative phase to evaluate its implementation in primary care. The RE-AIM implementation science framework (reach, adoption, implementation, efficacy, maintenance) guided both phases. Reporting followed the Standards for Reporting Implementation Studies (StaRI) guidelines.^13^ This study was approved by the University of Pennsylvania Institutional Review Board (Protocol #857597).

### Participants and recruitment

Participants included all nurses involved in triaging and treating UTI encounters at an urban academic family medicine practice consisting of six triage nurses, five nurse practitioners, and 20 physicians. In this practice, patients reporting UTI symptoms via telephone or patient portal are first triaged by a registered nurse, after which a nurse practitioner orders urine testing and prescribes antibiotics; physicians are rarely involved in this workflow. Nurses were engaged as partners throughout CDS development and evaluation. All participants provided verbal consent, no incentives were provided.

### Qualitative phase: intervention development

Workflow mapping and chart review were used to specify the evidence-practice gap using the AACTT (Actor, Action, Context, Target, and Time) behavior change framework: triage nurses (actor) need to systematically assess UTI-specific symptoms (action) during telephone or portal-based encounters (context) for all patients reporting possible UTI (target) before ordering urine testing or recommending antibiotics (time). Semi-structured interviews identified barriers to UTI triage and informed iterative refinement of the CDS using a user-centered approach.

Workflow mapping identified four specific gaps contributing to antibiotic overuse: incomplete assessment of UTI-specific symptoms, ordering urine cultures without assessing pyuria, limited use of non-antibiotic treatment options, and lack of patient counseling. Barriers and facilitators identified through semi-structured interviews were organized using the Theoretical Domains Framework (TDS) to inform selection of implementation strategies (see TIDieR checklist, Supplementary Table 1).

A multidisciplinary team (triage nurses, primary care clinicians, infectious disease specialists, urogynecologists) used these findings and evidence-based guidelines to develop a prototype EHR-integrated CDS. Symptom questions and scoring were adapted from validated questionnaires including the UTI Symptom Assessment Questionnaire, Acute Cystitis Symptom Score, and Lower Urinary Tract Dysfunction Research Network items.^14–16^

Semi-structured interviews were conducted in two phases using the same RE-AIM-informed interview guide with phase-specific probes (Appendix 1). Preimplementation interviews (n = 11) explored current triage workflow, barriers to symptom assessment, and CDS design preferences; findings informed iterative tool refinement through feedback sessions. The guide was pilot tested with three nurses.

### Intervention description

**Figure 1.**
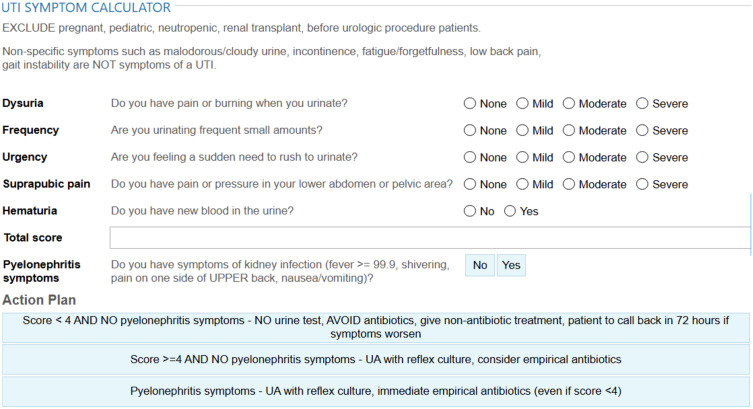
Nurse-led, EHR-integrated clinical decision support tool for outpatient UTI triage. A symptom score triggers algorithm-guided recommendations for testing, treatment, and counseling.

The final CDS consisted of a symptom score calculator and algorithm-based action plan embedded within the EHR triage documentation interface (Figure 1). Five cystitis symptoms (dysuria, frequency, urgency, suprapubic pain, hematuria), each scored 0–3, yield a total score of 0–15. The CDS also screens for pyelonephritis warning symptoms (flank pain, chills, fever >100.4°F, nausea, vomiting), allowing targeted reductions in testing and antibiotics for low-risk presentations while maintaining standard management for suspected pyelonephritis. A score <4 triggered recommendations for no urine testing or antibiotics; this conservative threshold was informed by nursing concerns about missing evolving infections identified during qualitative interviews.

Recommendations were displayed in real time as embedded text within the triage note upon completion of symptom entry. CDS use was optional; clinicians retained the ability to override recommendations based on clinical judgment. Nurses received a 15-minute training session during a staff meeting and written EHR-integrated guidance prior to implementation. No modifications were made to the CDS algorithm during the study period. Ongoing feedback during routine staff meetings addressed usability concerns and supported early adoption. A nurse champion was identified at the practice to facilitate peer support and troubleshoot workflow issues.

### Quantitative phase: implementation evaluation

The quantitative phase used a quasi-experimental pre–post design with interrupted time series (ITS) analysis. The study included a seven-month preimplementation period (July 2024–January 2025), CDS implementation in February 2025, and an eight-month postimplementation period (March–October 2025). Inclusion criteria were UTI encounters triaged by a nurse, identified using ICD-10 codes for acute cystitis (N30.00, N30.01, N30.90, N30.91, N39.0) and/or urine culture order. Exclusion criteria included pregnancy, urinary catheterization, and immunosuppression (transplant recipients, CD4 count < 200).

### Outcomes and measures

#### Data collection

Patient-level data extracted from the EHR included demographics and comorbidities. Encounter-level data included CDS use, documentation of UTI-specific symptoms, urine testing practices (urinalysis with microscopy, reflex urine culture, independent urine culture without microscopy), antibiotic prescribing, and patient counseling. Safety outcomes included pyelonephritis, urgent care or emergency department visits, and hospitalization within 30 days.

#### Implementation outcomes

The RE-AIM framework guided evaluation of implementation and clinical outcomes (Table 1). Reach (CDS use) was defined as the proportion of eligible UTI encounters with a documented CDS symptom score. Adoption was defined as the proportion of nurses using the CDS in ≥25% of eligible encounters, categorized as low (1%–24%), moderate (25%–49%), or high (≥50%). Implementation was operationalized as fidelity, usability, and acceptability. Postimplementation interviews (n = 11) assessed acceptability, usability, and perceived workflow impact. Maintenance was assessed as sustainability of clinical process outcomes over time.

**Table 1.** Implementation, clinical process, and safety outcomes defined using the RE-AIM framework Table 1 long description.

Domain	Definition and how measured
**Reach**	The proportion of the target population that participated in the intervention Proportion of eligible UTI encounters with documented CDS symptom score.
**Adoption**	Uptake of an intervention by intended end users Proportion of nurses using the CDS in ≥25% of eligible UTI encounters.
**Implementation**	The extent to which the intervention was implemented as intended
Fidelity	The extent to which the intervention was implemented as intended Proportion of postimplementation UTI encounters with symptom score<4 in which antibiotics were not prescribed.
Usability	Nurses’ perceived ease of use, efficiency, and workflow integration of CDS System Usability Scale (score 0–100, score ≥80 high usability)
Acceptability	The extent to which the intervention was perceived as satisfactory Qualitative interviews
**Efficacy**	Impact of the intervention on clinical process outcome and safety
Safety	Diagnosis of any of the following within 30 days of a UTI encounter Pyelonephritis ED/urgent care visits Hospitalization
Clinical Process Outcomes	1. Symptom documentation: proportion of UTI encounters with ≥2 documented UTI-specific symptoms
	2. Urine culture without microscopy
	3. Total antibiotics: Proportion of all UTI encounters prescribed an antibiotic
	4. Unnecessary antibiotic prescribing: proportion of antibiotic-treated encounters meeting ≥1 criterion: (A) asymptomatic bacteriuria, (B) negative pyuria (≤10 WBC/hpf), or (C) negative urine culture
	5. Proportion counseled on non-antibiotic treatment
**Maintenance**	Sustainability of intervention effects over time Interrupted time series analysis of symptom documentation, urine testing, and antibiotic prescribing.

#### Clinical process and safety outcomes

UTI-specific symptoms included dysuria, frequency, urgency, suprapubic pain, and hematuria; pyelonephritis symptoms included flank pain, chills, fever >100.4 °F (38°C), nausea, and vomiting.^17,18^ Asymptomatic bacteriuria was defined as a positive urine culture without documented UTI-specific symptoms.^6^ Unnecessary antibiotic prescribing was defined as treatment for asymptomatic bacteriuria, negative pyuria (≤10 WBC/hpf), or negative urine culture.^19,20^ Complete definitions are provided in Table 1.

### Sample size

Based on prior work,^17^ assuming baseline documentation of ≥2 UTI-specific symptoms of 49%, encounter-level CDS use of 60%, and a 60% relative increase in documentation with CDS use (yielding 66.6% postimplementation), approximately 122 encounters per period would provide 80% power (*α* = 0.05, two-sided) to detect a pre–post difference.

### Analysis

#### Qualitative

Methods and reporting followed the Consolidated Criteria for Reporting Qualitative Research (COREQ).^21^ Transcripts were reviewed for accuracy and analyzed using an inductive thematic analysis approach. Two investigators independently coded transcripts, iteratively refined a shared codebook, and resolved discrepancies through discussion and consensus. Codes were organized into themes through an iterative analytic process guided by the RE-AIM framework. Recruitment continued until thematic saturation was reached, defined as the point at which no new themes emerged in successive interviews.

#### Quantitative

Patient characteristics and implementation outcomes were compared between periods using t-tests for continuous variables and χ^2^ or Fisher’s exact tests for categorical variables. ITS analyses used monthly aggregated encounter rates, with February 2025 as the intervention start date. Segmented regression models included terms for baseline trend, level change at implementation, and postimplementation slope change; models used generalized linear regression with Newey-West standard errors to account for autocorrelation. Results are reported as level and slope changes with 95% confidence intervals. Two-sided *P* < .05 was considered significant. Analyses were performed using Stata/SE 18.0 (StataCorp).

## Results

### Qualitative results

Interviews with 11 triage nurses identified four barriers to effective UTI management: (1) incomplete symptom documentation due to time constraints and lack of standardized prompts; (2) reflexive urine testing regardless of symptoms; (3) medicolegal concerns about missing pyelonephritis, leading to defensive prescribing; and (4) difficulty managing patient pressure for antibiotics (Table 2). These findings informed CDS design: a structured symptom calculator ensured complete documentation, algorithm-guided reflex cultures reduced unnecessary testing, pyelonephritis screening addressed safety concerns, and standardized counseling language supported patient communication.

**Table 2.** Qualitative themes, theoretical domains framework (TDF) mapping, and illustrative quotes from nurse interviews Table 2 long description.

Theme	TDF domain(s)	Barrier description	Illustrative quote	CDS design feature addressing barrier
Incomplete symptom documentation	Environmental Context and Resources; Memory, Attention, and Decision Processes	Time constraints and lack of standardized prompts lead to inconsistent capture of UTI-specific symptoms	“There’s no set list of questions we’re supposed to ask -everyone does it differently, and when you’re rushed, you just ask the basics.”	Structured symptom calculator with standardized questions and automatic scoring
Reflexive urine testing	Behavioral Regulation; Social/Professional Role and Identity	Urine cultures ordered routinely regardless of symptom presentation, driven by established practice patterns	“It’s just what we do -if someone calls about a UTI, we send them for a culture. That’s always been the workflow.” [NP]	Algorithm-guided reflex culture ordering based on urinalysis results
Medicolegal concerns about missing pyelonephritis	Beliefs about Consequences; Emotion	Fear of missing serious infection leads to defensive testing and prescribing	“My biggest worry is that I’ll tell someone they don’t need antibiotics and then they end up in the hospital with a kidney infection.”	Explicit pyelonephritis screening questions with clear escalation pathways; conservative symptom score threshold (4)
Difficulty managing patient pressure	Social Influences; Skills	Patients expect antibiotics; nurses lack scripts for discussing non-antibiotic management	“Patients call expecting antibiotics. When you try to explain why they might not need them, they get upset and sometimes ask to speak to the doctor.”	Standardized counseling language providing evidence-based scripts for non-antibiotic management

Note: Quotes marked [NP] are from nurse practitioners; unmarked quotes are from triage nurses. CDS, clinical decision support; TDF, Theoretical Domains Framework; UTI, urinary tract infection.

### Quantitative results

#### Patient characteristics

Patient characteristics were similar between preimplementation (n = 133) and postimplementation (n = 125) periods, with no significant differences in age, sex, insurance type, or comorbidities (Table 3). Among postimplementation encounters with CDS scores, median individual symptom scores were 1–2 (IQR 0–2), indicating mild-to-moderate severity; median total symptom score was 5.5 (IQR 4–8; range 0–15). Notably, rates of pyuria (44% vs 49%) and positive urine culture (51% vs 56%) were similar between encounters with symptom scores ≥4 versus <4.

**Table 3.** Patient characteristics in pre and postimplementation periods Table 3 long description.

Characteristic	Pre-implementation (n = 133)	Post-implementation (n = 125)	*P*-value
Age (years), mean (SD)	46.6 (15.4)	45.1 (16.3)	.46
Sex, n (%)			.81
Male	14 (11)	12 (10)	
Female	119 (89)	113 (90)	
Preferred language, n (%)			.49
English	130 (98)	120 (96)	
Other	3 (2)	5 (4)	
Race, n (%)			.002^a^
White	15 (11)	17 (14)	
Black	107 (80)	90 (72)	
Other/mixed	4 (3)	17 (14)	
Unknown	7 (5)	1 (1)	
Hispanic or Latino, n (%)	7 (5)	6 (5)	.87
Insurance type, n (%)			.40
Private	73 (55)	75 (60)	
Medicare (traditional)	10 (8)	7 (6)	
Medicare advantage	11 (8)	11 (9)	
Low-income medicare	0 (0)	0 (0)	
Medicaid	34 (26)	32 (26)	
Uninsured/other	5 (4)	0 (0)	
Any kidney diagnosis, n (%)	15 (11)	21 (17)	.19
Diabetes, n (%)	19 (14)	30 (24)	.05^a^
Catheter use, n (%)	2 (2)	3 (2)	.68

a Race and diabetes prevalence differed between periods; these differences were not associated with primary outcomes in sensitivity analyses.

#### Implementation outcomes

Reach was 60.8% (76/125 nurse-triaged UTI encounters with documented CDS symptom scores). All 11 nurses met adoption criteria (2 moderate, 9 or 82% high adopters). Usability was good, with median System Usability Scale score of 81 (IQR 65–90); participants rated the CDS as easy to use and well-integrated into workflow. Fidelity, defined as adherence to algorithm recommendations for no antibiotics when score <4, was 77.8% (14/18). Among the four encounters with score <4 in which antibiotics were prescribed, chart review indicated patient request (n = 2) and clinician-initiated urine testing (n = 2).

#### Clinical process and safety outcomes

Documentation of ≥2 UTI-specific symptoms increased significantly from 73% to 87% postimplementation (*P* = .004), with similar improvements in individual symptom and pyelonephritis symptom documentation (Table 4). Urine testing practices shifted toward guideline-concordant ordering: urinalysis with microscopy increased (53% to 65%), reflex urine culture increased (0% to 47%, *P* < .001), and independent urine culture without microscopy decreased (47% to 23%, *P* < .001). Patient counseling on non-antibiotic treatment increased from 18% to 55% (*P* < .001).

**Table 4.** Clinical process and safety outcomes before and after CDS implementation Table 4 long description.

UTI relevant variables	Preimplementation period (n = 133)	Postimplementation period (n = 125)	*P*-value
**Symptom outcomes**			
≥2 UTI symptoms documented	97 (73)	109 (87)	.004
Pyelonephritis suspected	13 (10)	23 (18)	.046
**Urine testing outcomes**			
Urinalysis with microscopy	70 (53)	80 (65)	.053
Urinalysis with reflex urine culture	0 (0)	59 (47)	<.001
Independent urine culture without microscopy	62 (47)	29 (23)	<.001
Asymptomatic bacteriuria	12 (9)	2 (2)	.014
**Safety outcomes**			
Pyelonephritis diagnosis within 30 days	0 (0)	1 (1)	.482
ED or urgent care visit within 30 days	2 (2)	3 (2)	.675
Hospital admission for UTI within 30 days	0	0	
**Antibiotic outcomes**			
Total antibiotics prescribed	75 (56)	60 (48)	.199
Unnecessary antibiotic prescribing^a^	39 (52)	24 (40)	.165
A. Asymptomatic bacteriuria	15 (20)	2 (3)	.003
B. Negative pyuria	15 (20)	12 (20)	.962
C. Negative urine culture	22 (29)	18 (30)	.982
**Patient counseling**			
Patient received UTI education	1 (1)	37 (30)	<.001
Patient counseled on non-antibiotic treatment	24 (18)	69 (55)	<.001
Patient received prevention counseling	12 (9)	43 (35)	<.001

a Unnecessary antibiotic prescribing defined as meeting ≥1 criterion (A, B, or C); denominator is antibiotic-treated encounters; criteria are not mutually exclusive.

All values are n (%) unless otherwise specified. Symptom documentation reflects presence documented in the EHR; absence of documentation was treated as absence of symptom.

Antibiotic prescribing for asymptomatic bacteriuria decreased significantly (20% vs 3%, *P* = .003) (Table 4). Total antibiotic prescribing and unnecessary antibiotic prescribing also declined, though these differences were not statistically significant.

Rates of pyelonephritis, urgent care/ED visits, and hospitalizations were low and similar between periods. Among the 14 patients with low symptom scores who did not receive antibiotics as recommended, none experienced adverse outcomes within 30 days (0/14; 95% CI, 0%–19.3%).

#### Sustainability

**Figure 2.**
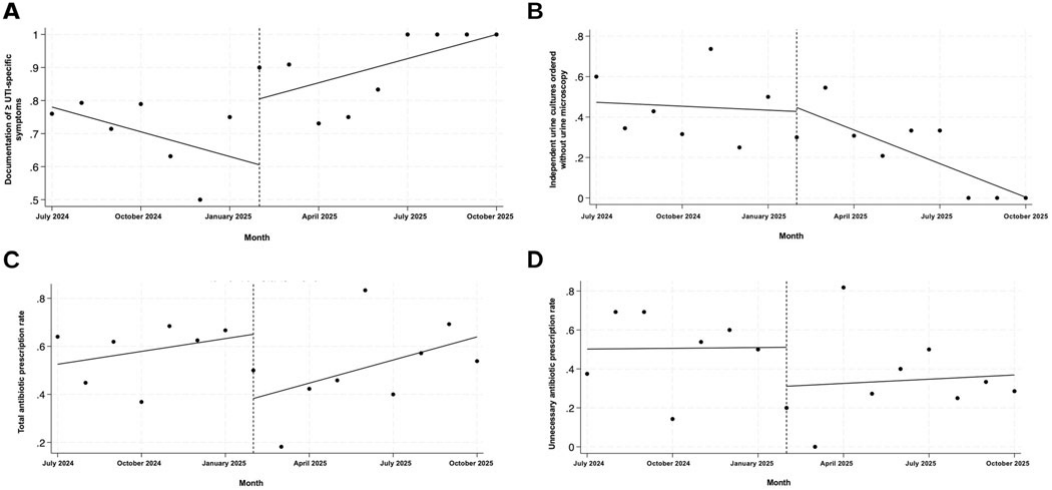
Interrupted time series analysis of clinical process and antibiotic outcomes following CDS implementation. Monthly rates of (A) documentation of ≥2 UTI-specific symptoms, (B) urine culture ordering without microscopy, (C) total antibiotic prescribing, and (D) unnecessary antibiotic prescribing. Dots represent observed rates; lines represent model-predicted values. The vertical dashed line indicates CDS implementation (February 2025). Detailed results in Table 5.

Interrupted time series analysis showed that documentation of ≥2 UTI-specific symptoms increased significantly at implementation (level change + 20.0%; 95% CI, 0.2–39.7), with a significant change in trend (slope change + 4.9%/month; 95% CI, 0.7–9.2) and sustained improvement postimplementation (slope + 2.4%/month; 95% CI, 0.1–4.7) (Figure 2, Table 5). Ordering urine culture without microscopy showed a significant change in trend (slope change −4.9%/month; 95% CI, −8.8 to −1.1) with sustained reduction postimplementation (slope −5.5%/month; 95% CI, −7.9 to −3.2). Total antibiotic prescribing showed a significant immediate decrease at implementation (level change −26.7%; 95% CI, −45.6 to −7.8) but no significant change in trend. Unnecessary antibiotic prescribing showed no significant changes.

**Table 5.** Interrupted time series analysis of sustainability of clinical process outcomes after CDS implementation Table 5 long description.

Outcome	Baseline (%)	Preintervention trend (%/month) (95% CI)	Immediate level change at implementation (%/month) (95% CI)	Change in trend postimplementation (%/month) (95% CI)	Postimplementation trend (%/month) (95% CI)
Documentation of ≥2 UTI symptoms	78.0	−2.5 (−5.4 to 0.5)	+20.0 (0.2 to 39.7)	+4.9 (0.7 to 9.2)	+2.4 (0.1 to 4.7)
Urine culture without microscopy ordered	47.3	−0.6 (−4.3 to 3.0)	+ 1.9 (−20.5 to 24.4)	−4.9 (−8.8 to −1.1)	−5.5 (−7.9 to −3.2)
Total antibiotics prescribed	52.5	+1.8 (−0.8 to 4.4)	−26.7 (−45.6 to −7.8)	+1.4 (−2.6 to 5.5)	+3.2 (0.4 to 6.0)
Unnecessary antibiotics prescribed	50.2	+0.1 (−4.2 to 4.5)	−20.0 (−53.8 to 13.8)	+0.6 (−6.0 to 7.2)	+0.7 (−4.2 to 5.7)

## Discussion

Prior CDS interventions for UTI stewardship have focused primarily on physician prescribers and antibiotic selection - such as reducing fluoroquinolone or extended-spectrum antibiotic use - rather than upstream triage processes.^22–24^ This study evaluated a nurse-led, EHR-integrated CDS tool designed through user-centered methods to standardize symptom assessment before testing or treatment decisions. By embedding structured symptom assessment within nurse triage workflows, this intervention introduced a deliberate pause that interrupted routine test ordering and promoted more thoughtful diagnostic sequencing.

### Mechanisms of success: linking barriers to solutions

The success of this intervention reflects explicit alignment between qualitatively identified barriers and CDS design features (Table 2). Incomplete symptom documentation was addressed through a structured calculator ensuring consistent capture; routine urine testing was countered by algorithm-guided reflex culture ordering, shifting practice from independent culture without microscopy (47% to 23%) to urinalysis-guided testing (0% to 47%). Medicolegal concerns about missing pyelonephritis were addressed through explicit screening questions and a conservative symptom threshold (<4), likely facilitating clinician trust. Difficulty managing patient pressure for antibiotics was mitigated through standardized counseling language; the increase in non-antibiotic counseling (18% to 55%) suggests active utilization. These findings demonstrate the value of theory-informed intervention development using implementation science frameworks to enhance adoption and sustainability.

### Critical success factors for CDS implementation

Several design features aligned with established predictors of CDS effectiveness. Automatic provision within the existing triage workflow minimized cognitive burden, a critical success factor for CDS adoption.^25^ Early and sustained nursing involvement may have contributed to high adoption (82%), exceeding previously reported rates (∼30%) for similar interventions.^26^ Fidelity was also high (77.8%); the four encounters with antibiotics prescribed contrary to recommendations reflected contextual concerns (patient request, clinician-initiated testing) rather than lack of trust in the algorithm.

### Comparison with prior work

Several questionnaires have been developed to diagnose UTI.^15,16^ In contrast, the purpose of our CDS symptom score calculator and algorithm is not to diagnose infection but to guide triage decisions - specifically, to identify patients with minimal symptom burden who can safely forgo immediate testing and antibiotics. Microbiologic findings did not differ by symptom score category, and rates of pyuria and positive urine cultures were substantial even among patients with low scores; this lack of correlation reinforces the rationale for symptom-based rather than culture-based decision-making. This approach aligns with recently published expert consensus recommendations. Meddings et al developed a symptom-based algorithm for outpatient UTI triage that recommends against urine testing or antibiotics for nonspecific symptoms such as changes in urine color or odor alone.^11^ The American Urogynecologic Society and Society of Urodynamics, Female Pelvic Medicine and Urogenital Reconstruction has also endorsed a symptom-based approach.^27^ Notably, these recommendations have not yet been evaluated for clinical outcomes; the present study provides early implementation evidence supporting the feasibility and safety of this approach.

### Clinical implications

Implementation of the CDS was not associated with increased rates of pyelonephritis, emergency department visits, urgent care visits, or hospitalizations within 30 days. Among the 14 patients with low symptom scores who did not receive antibiotics, none experienced adverse outcomes. These safety findings are consistent with population-based data suggesting that pyelonephritis occurs in only 1%–1.5% of uncomplicated UTIs, even when up to 50% are managed without antibiotics.^8,9^ The absence of safety signals supports the clinical validity of using structured symptom assessment to safely defer antibiotics in patients with low symptom burden.

The marked reduction in antibiotic treatment of asymptomatic bacteriuria (20% vs 3%) represents a clinically meaningful improvement in stewardship practice achieved without compromising patient safety. Although overall antibiotic prescribing did not significantly decline, the early implementation phase appropriately prioritized clinician trust and adoption. The combination of high fidelity to algorithm recommendations (77.8%) and absence of adverse safety outcomes suggests that nurses and clinicians were able to confidently apply the CDS to defer antibiotics when clinically appropriate.

### Persistent barriers and postimplementation plans

Postimplementation interviews identified persistent barriers, including uncertainty about CDS use when non-UTI symptoms were prominent or in male patients, highlighting opportunities for clearer eligibility guidance. As concerns about missing pyelonephritis decreased with experience, ongoing collaboration with nurses led to development of a revised algorithm that encourages initial non-antibiotic management with antibiotics reserved for non-responders (Supplementary Figure 1). This revised algorithm is currently being evaluated at our institution.

### Next steps, generalizability, and scalability

Next steps include prospective evaluation of the revised algorithm and cost-effectiveness analysis to quantify potential savings from reduced unnecessary testing and antibiotic prescribing. Potential barriers include EHR integration capabilities, nurse training infrastructure, and institutional support for nurse-led protocols. Facilitators include the generalizable algorithm structure adaptable to other EHR platforms, use of validated symptom questionnaires, and the RE-AIM framework providing a replicable evaluation approach. The TIDieR checklist (Supplementary Table 1) provides detailed intervention specifications to support adaptation and replication in other settings.

### Strengths and limitations

Strengths include user-centered development informed by qualitative identification of workflow barriers, mixed-methods evaluation linking implementation with clinical process outcomes, and focus on nursing triage as a high-leverage point for outpatient stewardship. ITS analysis, a rigorous quasi-experimental design controlling for secular trends, provides stronger causal inference than simple prepost comparison.

Several limitations should be noted. The study was conducted at a single academic family medicine practice using Epic EHR, which may limit generalizability to settings with different staffing models, workflows, or EHR platforms. Sample size was modest and powered to detect changes in symptom documentation rather than antibiotic prescribing; larger studies over longer periods are needed to confirm effects on antibiotic outcomes. Outcomes relied on EHR documentation, which may not fully capture clinical decision-making. Physician perspectives on the CDS were not assessed, which may limit understanding of interdisciplinary dynamics influencing prescribing decisions. We did not formally evaluate optimal symptom score thresholds; prior studies deriving diagnostic thresholds compared younger women with acute cystitis to controls without cystitis,^15^ which may not apply to primary care triage populations with higher rates of asymptomatic bacteriuria.

### Conclusions

Standardizing symptom assessment through nurse-led, EHR-integrated clinical decision support was associated with reduced antibiotic prescribing for asymptomatic bacteriuria without evidence of short-term safety concerns. By leveraging the central role of nurses in outpatient UTI triage and systematically addressing workflow barriers identified through qualitative research, this approach embeds antibiotic stewardship at an early point in the care pathway. Future work should focus on confirming safety in larger populations, optimizing symptom score thresholds, and conducting formal cost-effectiveness analysis to support broader dissemination.

## Supporting information

10.1017/ash.2026.10772.sm001 Qin et al. supplementary material 1 Qin et al. supplementary material

10.1017/ash.2026.10772.sm002 Qin et al. supplementary material 2 Qin et al. supplementary material

## Data Availability

Data are available upon reasonable request to the corresponding author.

## Supplementary material

The supplementary material for this article can be found at https://doi.org/10.1017/ash.2026.10772.

## Table 1 Long description

Table with two columns: Domain and Definition and how measured. The table lists various domains such as Reach, Adoption, Implementation, Usability, Acceptability, Efficacy, Safety, Clinical Process Outcomes, and Maintenance. Each domain includes a definition and how it is measured. For example, Reach is defined as the proportion of eligible UTI encounters with a documented CDS symptom score. Adoption is defined as the proportion of nurses using the CDS in 25% of eligible encounters, categorized as low, moderate, or high. Implementation includes fidelity, usability, and acceptability, with specific measures for each. Usability is measured using the System Usability Scale. Acceptability is assessed through qualitative interviews. Efficacy, Safety, and Clinical Process Outcomes include specific clinical process and safety measures. Maintenance is assessed as the sustainability of clinical process outcomes over time.

Navigate back to Table 1.

## Table 2 Long description

A table comparing barriers to UTI management and CDS design features addressing these barriers. The table has four rows and four columns. The columns are labeled Theme, TDF domain(s), Barrier description, Illustrative quote, and CDS design feature addressing barrier. Row 1: Theme, Incomplete symptom documentation; TDF domain(s), Environmental Context and Resources; Memory, Attention, and Decision Processes; Barrier description, Time constraints and lack of standardized prompts lead to inconsistent capture of UTI-specific symptoms; Illustrative quote, “There’s no set list of questions we’re supposed to ask - everyone does it differently, and when you’re rushed, you just ask the basics.”; CDS design feature addressing barrier, Structured symptom calculator with standardized questions and automatic scoring. Row 2: Theme, Reflexive urine testing; TDF domain(s), Behavioral Regulation; Social/Professional Role and Identity; Barrier description, Urine cultures ordered routinely regardless of symptom presentation, driven by established practice patterns; Illustrative quote, “It’s just what we do - if someone calls about a UTI, we send them for a culture. That’s always been the workflow.” [NP]; CDS design feature addressing barrier, Algorithm-guided reflex culture ordering based on urinalysis results. Row 3: Theme, Medicolegal concerns about missing pyelonephritis; TDF domain(s), Beliefs about Consequences; Emotion; Barrier description, Fear of missing serious infection leads to defensive testing and prescribing; Illustrative quote, “My biggest worry is that I’ll tell someone they don’t need antibiotics and then they end up in the hospital with a kidney infection.”; CDS design feature addressing barrier, Explicit pyelonephritis screening questions with clear escalation pathways; conservative symptom score threshold (4). Row 4: Theme, Difficulty managing patient pressure; TDF domain(s), Social Influences; Skills; Barrier description, Patients expect antibiotics; nurses lack scripts for discussing non-antibiotic management; Illustrative quote, “Patients call expecting antibiotics. When you try to explain why they might not need them, they get upset and sometimes ask to speak to the doctor.”; CDS design feature addressing barrier, Standardized counseling language providing evidence-based scripts for non-antibiotic management.

Navigate back to Table 2.

## Table 3 Long description

The table compares patient characteristics between preimplementation (n = 133) and postimplementation (n = 125) periods. It has 13 rows and 4 columns. The columns are labeled Characteristic, Pre-implementation, Post-implementation, and P-value. The rows include Age (years), mean (SD), Sex, n (%), Preferred language, n (%), Race, n (%), Hispanic or Latino, n (%), Insurance type, n (%), Any kidney diagnosis, n (%), Diabetes, n (%), and Catheter use, n (%). Each row provides specific data for preimplementation and postimplementation periods, along with P-values indicating statistical significance. Notable trends include significant differences in race (P-value = 0.002) and diabetes (P-value = 0.05).

Navigate back to Table 3.

## Table 4 Long description

The table compares urinary tract infection (UTI) relevant variables during preimplementation and postimplementation periods. It has 5 columns: UTI relevant variables, Preimplementation period (n=133), Postimplementation period (n=125), and P-value. The table is divided into sections: Symptom outcomes, Urine testing outcomes, Safety outcomes, Antibiotic outcomes, and Patient counseling. Each section lists specific variables with corresponding values for both periods and P-values indicating statistical significance. For example, under Symptom outcomes, ≥2 UTI symptoms documented increased from 97 (73 percent) to 109 (87 percent) with a P-value of .004. Under Urine testing outcomes, Urinalysis with microscopy increased from 70 (53 percent) to 80 (65 percent) with a P-value of .053. Under Patient counseling, Patient received UTI education increased from 1 (1 percent) to 37 (30 percent) with a P-value of <.001.

Navigate back to Table 4.

## Table 5 Long description

A table with five rows and five columns comparing the impact of clinical decision support system implementation on various outcomes related to urinary tract infection management. The columns are labeled Baseline (percent), Preintervention trend (percent per month with 95 percent confidence interval), Immediate level change at implementation (percent per month with 95 percent confidence interval), Change in trend postimplementation (percent per month with 95 percent confidence interval), and Postimplementation trend (percent per month with 95 percent confidence interval). The rows are labeled Documentation of 2 or more UTI symptoms, Urine culture without microscopy ordered, Total antibiotics prescribed, and Unnecessary antibiotics prescribed. Each row provides specific values for each column, indicating the changes in trends and levels at different stages of the implementation.

Navigate back to Table 5.
