# Supplementary figures and images for: Development and implementation of a nurse-led clinical decision support tool for urinary tract infection
Source: Antimicrob Steward Healthc Epidemiol. 2026 Jul 6;6(1):e200. doi: 10.1017/ash.2026.10772 (PMC13343335; doi:10.1017/ash.2026.10772)

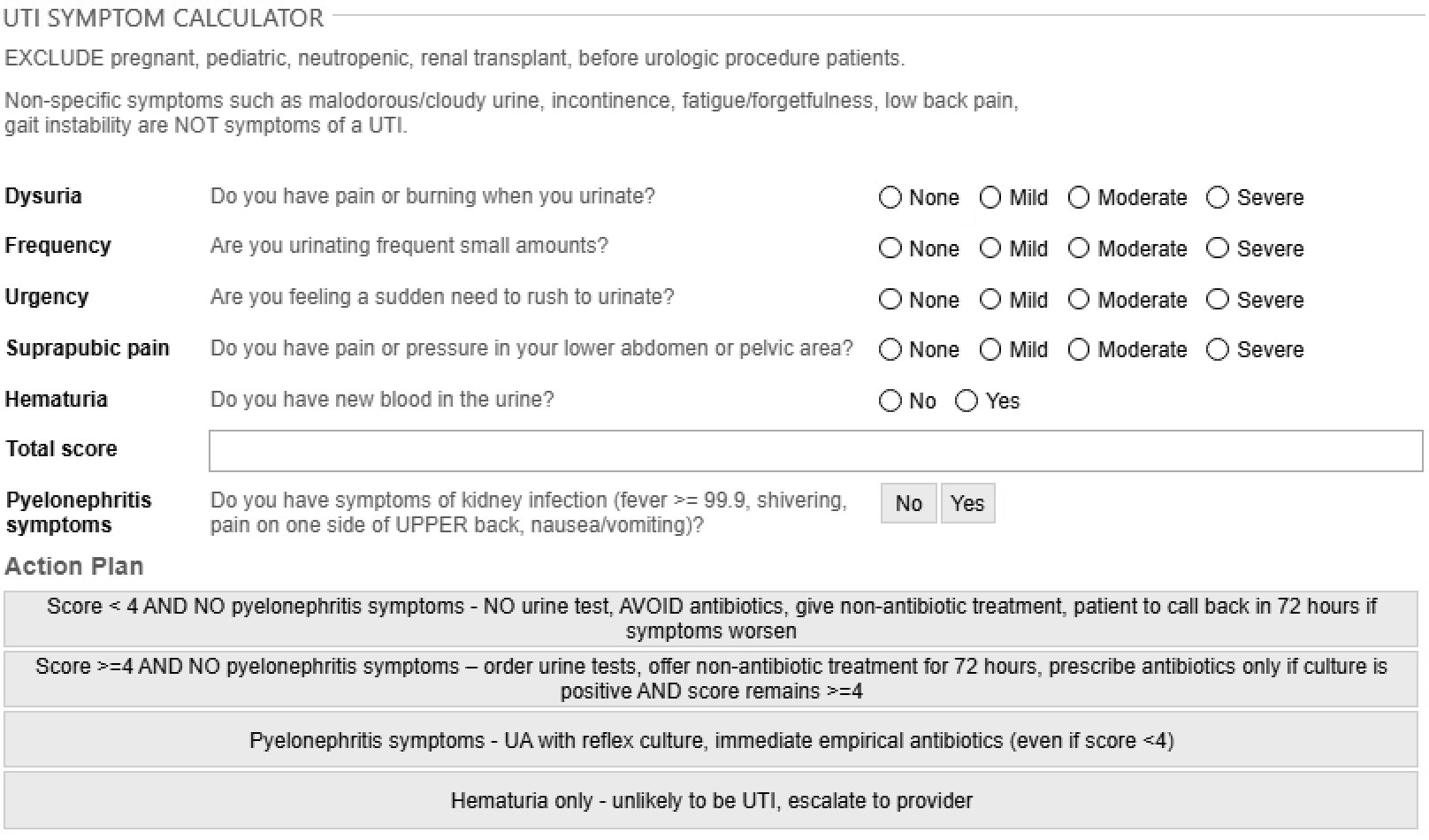

Supplement: Qin et al. supplementary material 1 — Qin et al. supplementary material [file S2732494X26107724sup001.tiff]
