# Supplementary material for: Development and implementation of a nurse-led clinical decision support tool for urinary tract infection
Source: Antimicrob Steward Healthc Epidemiol. 2026 Jul 6;6(1):e200. doi: 10.1017/ash.2026.10772 (PMC13343335; doi:10.1017/ash.2026.10772)
Supplement: Qin et al. supplementary material 2 — Qin et al. supplementary material [file S2732494X26107724sup002.docx]

**Supplementary Table 1. Template for Intervention Description and Replication (TIDieR) Checklist**

| **Item No.** | **Item** | **Description for This Study** | **Location in Manuscript** |
| --- | --- | --- | --- |
| 1 | **Brief name** | Nurse-led, EHR-integrated clinical decision support (CDS) tool for outpatient UTI symptom triage | Title, Abstract, Methods |
| 2 | **Why (rationale)** | To address evidence-practice gaps in UTI management: incomplete symptom assessment, reflexive urine testing, treatment of asymptomatic bacteriuria, and lack of patient counseling. Qualitative work identified four barriers mapped to Theoretical Domains Framework constructs (Table 2). The intervention aimed to standardize symptom assessment during nurse triage to reduce unnecessary testing and antibiotic prescribing. | Introduction, Methods (Qualitative Phase), Table 2 |
| 3 | **What (materials)** | (1) EHR-embedded symptom score calculator with 5 cystitis symptoms (dysuria, frequency, urgency, suprapubic pain, hematuria), each scored 0–3 (total 0–15); (2) Pyelonephritis screening questions (flank pain, chills, fever >100.4°F, nausea, vomiting); (3) Algorithm-based action plan with threshold-driven recommendations; (4) Standardized patient counseling messages for non-antibiotic management. Symptom questions adapted from validated instruments: UTI Symptom Assessment Questionnaire, Acute Cystitis Symptom Score, and LURN SI-29. | Methods (Intervention Description), Figure 1, References 15–17 |
| 4 | **What (procedures)** | During telephone or patient portal-based encounters for possible UTI, triage nurses complete the CDS symptom assessment. The CDS automatically calculates a symptom score and displays algorithm-guided recommendations: score 4 triggers recommendation for no urine testing or antibiotics; score ≥4 triggers urinalysis with reflex culture. Pyelonephritis symptoms trigger escalation. Nurses deliver standardized counseling for non-antibiotic management when appropriate. Clinicians retain ability to override recommendations. | Methods (Intervention Description, Implementation Strategy), Figure 1 |
| 5 | **Who provided** | Registered nurses (n=6) and nurse practitioners (n=5) performing UTI triage at an urban academic family medicine practice. Nurses received brief training during staff meetings and through written EHR-integrated guidance prior to implementation. | Methods (Participants, Implementation Strategy) |
| 6 | **How (mode of delivery)** | The CDS was delivered electronically via the Epic EHR system, embedded within the existing nurse triage documentation interface. The intervention was integrated into routine telephone and patient portal-based encounters (not face-to-face). Recommendations were displayed in real time upon completion of symptom assessment. | Methods (Implementation Strategy) |
| 7 | **Where** | Single urban academic family medicine practice affiliated with the University of Pennsylvania Health System, Philadelphia, USA. The practice includes 6 triage nurses, 5 nurse practitioners, and 20 physicians. | Methods (Study Design and Setting, Participants) |
| 8 | **When and how much** | The CDS was available for use during all nurse-triaged UTI encounters beginning February 2025. Symptom assessment required approximately 2–3 minutes per encounter. The post-implementation evaluation period was 8 months (March–October 2025). Reach was 60.8% (76/125 eligible encounters with documented CDS use). | Methods (Quantitative Phase), Results (Implementation Outcomes) |
| 9 | **Tailoring** | The symptom score threshold (4) was set conservatively based on nursing concerns about missing evolving infections identified during qualitative interviews. The CDS allowed clinician override of recommendations based on clinical judgment. Standardized counseling messages were developed to address nurse-identified barriers in managing patient expectations. | Methods (Intervention Description), Results (Qualitative Results), Discussion |
| 10 | **Modifications** | No modifications were made to the CDS algorithm during the 8-month post-implementation period. Post-implementation interviews identified opportunities for refinement (e.g., clearer eligibility guidance for mixed-symptom presentations, male patients). A revised algorithm encouraging initial non-antibiotic treatment is under development for future testing (Supplementary Figure 1). | Discussion (Persistent Barriers and Postimplementation Plans) |
| 11 | **How well (planned)** | Fidelity was planned to be assessed as adherence to algorithm recommendations (i.e., proportion of encounters with score 4 in which antibiotics were not prescribed). Implementation outcomes were evaluated using the RE-AIM framework: Reach (proportion of encounters with CDS use), Adoption (proportion of nurses using CDS in ≥25% of encounters), Implementation (fidelity, usability via System Usability Scale, acceptability via qualitative interviews), and Maintenance (sustainability of outcomes over time via interrupted time series). | Methods (Outcomes and Measures), Table 1 |
| 12 | **How well (actual)** | Fidelity: 77.8% (14/18 encounters with score 4 followed recommendation for no antibiotics). Among 4 non-adherent encounters, reasons were patient request (n=2) and clinician-initiated testing (n=2). Reach: 60.8%. Adoption: 100% of nurses met adoption criteria; 82% were high adopters (≥50% of encounters). Usability: Median System Usability Scale score 81 (IQR 65–90). Maintenance: Interrupted time series showed sustained improvements in symptom documentation and reductions in urine culture ordering without microscopy over 8 months. | Results (Implementation Outcomes, Sustainability), Table 5, Figure 2 |

Abbreviations: CDS, clinical decision support; EHR, electronic health record; IQR, interquartile range; LURN SI-29, Lower Urinary Tract Dysfunction Research Network Symptom Index-29; RE-AIM, Reach, Effectiveness, Adoption, Implementation, Maintenance; UTI, urinary tract infection.

This checklist was completed following the Template for Intervention Description and Replication (TIDieR) guidelines.^1^
